# Xanthohumol ameliorates diabetic kidney disease through suppression of renal fibrosis by regulating SNHG10/miR-378b

**DOI:** 10.3389/fphar.2025.1532517

**Published:** 2025-05-27

**Authors:** Jian Hao, Hui Li, Weimin Yu

**Affiliations:** Department of Nephrology, Shanxi Bethune Hospital, Shanxi Academy of Medical Sciences, Tongji Shanxi Hospital, Third Hospital of Shanxi Medical University, Taiyuan, China

**Keywords:** xanthohumol, diabetic nephropathy, renal fibrosis, SNHG10/miR-378b, interstitial fibrosis, inflammatory response

## Abstract

**Content:**

Diabetic kidney disease (DKD), commonly termed diabetic nephropathy (DN), is characterized by oxidative stress and renal tubular epithelial cells apoptosis driven by high glucose (HG).

**Objective:**

To explore the protective effects and underlying mechanism of xanthohumol in DN mice and HG-induced HK-2 cells.

**Materials and methods:**

The STZ-treated mice and HG stimulated HK-2 cells were applied to establish *in vivo* and *in vitro* DN models. The concentrations of blood glucose, serum creatinine, BUN and urine creatinine, and β-n-acetylglucosaminidase (NAG) activity was determined. The pathological changes of renal tissues were evaluated by Masson and periodic acid schiff (PAS) staining. TNF-α, IL-1β and IL-6 levels were detected using ELISA. Furthermore, CCK-8 assay and flow cytometer analysis were applied for determining HK-2 cells viability and apoptosis, respectively. Gene and protein levels was evaluated by qRT-PCR analysis and western blot/IHC. The relationship between lncRNA SNHG10 and miR-378b was confirmed by luciferase reporter assay.

**Results:**

Xanthohumol effectively improves DN-stimulated kidney structural and functional abnormalities. LncRNA SNHG10 was downregulated in the renal tissues of DN mice and HG induced HK-2 cells, while this inhibition was reversed by xanthohumol treatment. We also noted that xanthohumol remarkably reversed HG induced HK-2 cells injury. Upregulation of lncRNA SNHG10 also improved DN in mice. Meanwhile, downregulation of SNHG10 reversed the effects of xanthohumol on HG-induced HK-2 cells. Additionally, miR-378b directly targeted lncRNA SNHG10.

**Conclusion and discussion:**

Xanthohumol inhibited the progression of DN by regulating SNHG10/miR-378b, indicating a novel understanding of xanthohumol in DN progression and providing a latent therapeutic target for DN therapy.

## Introduction

Diabetic kidney disease (DKD), commonly termed diabetic nephropathy (DN), a severe complication of diabetes, is a common cause of chronic kidney disease (Hostetter, 1985). The typical pathological features of DN include glomerular and tubular hypertrophy, glomerular basement membrane thickening, inflammatory infiltration and renal interstitial fibrosis (Hu et al., 2023; Jiang et al., 2020). Increasing evidence has shown that continuous high glucose environment, oxidative stress and inflammatory response induce cell death of renal tubular epithelial cells (RTECs), which plays a key role in the occurrence and development of DN (Chen et al., 2022; Wang et al., 2022). However, due to the multiple influencing factors and complex process, the pathogenesis of DN is still unclear, and there are no effective prevention and control measures currently. At present, the treatment and management strategies of DN mainly focus on reducing body weight, blood glucose and pressure (Tang et al., 2021), and the common first-line treatment of DN is renin-angiotensin system inhibitors (Ishibashi et al., 2023). Therefore, a better understanding of the pathogenesis of DN and the search for new biomarkers are still urgently needed.

Xanthohumol is a natural pentenyl flavonoid compound, also known as hops, isolated from the female inflorescence of Humulus lupus (Girisa et al., 2021). Recently, increasing evidences have shown that multiple cellular targets of xanthohumol have been identified in human cancer cells, including non-small cell lung cancer (Zhao et al., 2023), colon cancer (Torrens-Mas et al., 2022) and breast cancer (Sun et al., 2018). Xanthohumol has also been reported to restore hepatic glucolipid metabolism balance in type 1 diabetic rat models (Lima-Fontes et al., 2017). Xanthohumol can reduce type 2 diabetes-associated oxidative stress by downregulating galectin-3 (Luís et al., 2018). Ma et al. suggested that xanthohumol protects cognitive performance in diabetic model rats by inhibiting protein kinase B/nuclear factor kappa-B pathway (Ma et al., 2021b). Costa et al. revealed that xanthohumol can ameliorate diabetic-related metabolic dysfunctions in mice (Costa et al., 2017). Wang et al. confirmed that xanthohumol alleviates type II diabetes mellitus-induced liver steatosis and fibrosis by mediating the NRF2/RAGE/NF-kappaB signaling pathway (Wang et al., 2021). Furthermore, Li et al. have found the protective effect of xanthohumol on human renal cells and in STZ-induced DN mice by activating Nrf2 signaling pathway, suggesting that xanthohumol might serve as a promising therapeutic drug candidate for DN treatment (Li et al., 2023). A recent study indicated that xanthohumol attenuates hyperglycemia-induced renal tubular injury potentially through the inhibition of TXNIP expression (Zhang et al., 2025). However, the therapeutic effect and mechanism of xanthohumol in DN remain to be explored.

Long noncoding RNAs (lncRNAs) are a class of non-coding RNAs with a length of over 200 nucleotides, which regulate gene expression by acting as microRNAs sponges (Wu et al., 2023; Nojima and Proudfoot, 2022). LncRNAs are thought to be involved in tumor growth and metastasis through a variety of molecular mechanisms, including breast cancer (Zhang et al., 2023a; b), gastric cancer (Gong et al., 2023) and non-small cell lung cancer (He et al., 2021). Increasing evidences have revealed that lncRNAs are associated with the development of DN, such as lncRNA SOX2OT (Chen et al., 2021), lncRNA NEAT1 (Li et al., 2020a) and lncRNA XIST (Long et al., 2023). Therefore, it is important to explore the expression form and potential mechanism of lncRNAs in DN to understand the pathogenesis of DN. Small nucleolar RNA host gene 10 (SNHG10), a newly identified long non-coding RNA, has been reported to promote the osteosarcoma proliferation and invasion by wnt/beta-catenin signaling (Zhu et al., 2020). In addition, lncRNA SNHG10 upregulates BIN1 to suppress the tumorigenesis and epithelial-mesenchymal transition of epithelial ovarian cancer through sponging miR-200a-3p (Lv et al., 2022). However, the role of SNHG10 in DN remains unclear. Bioinformatics analysis showed that miR-378b is the target gene of SNHG10. miR-378b has been shown to be involved in the regulation of insulin sensitivity (Li et al., 2020b), and it plays an important regulatory role in organ fibrosis (Hyun et al., 2016). Therefore, we hypothesize that SNHG10 may play a role in DN by regulating miR-378b.

Thus, our investigation was designed to determine whether xanthohumol exerted its protective effect in DN by regulating the expression of lncRNA SNHG10 and explore the molecular regulatory mechanism. Our resulting findings provide new insights into the clinical treatment of DN.

## Materials and methods

### Establishment of animal models and treatment

Male C57BL/6J mice (7 weeks old) were acquired from Hubei Beiente Biotechnology Co., Ltd. All mice were cultured in a specific pathogen-free environment with a standard light-dark cycle of 25°C for 12 h and had free access to food and water. The DN mouse model was established according to a previous study (Lv et al., 2023). Mice were fed a high-fat diet for 2 months, and then streptozotocin (STZ; 50 mg/kg) was injected intraperitoneally for 7 consecutive days to induce DN mouse model. Seven days after injection, C57BL/6J mice were monitored for blood glucose levels. The control group of mice continued to be fed a standard diet. Then mice were intraperitoneally injected with 25 mg/kg of Xanthohumol, control-plasmid or SNHG10-plasmid, and blood, urine, and renal tissues were collected for the following experiments. The animal experiments were approved by the Ethics Review Committee of Bestcell Model Biological Center (approval number 2024-01-29A).

### Cell cultures and treatment

The HK-2 cells were acquired from ATCC and cultured in DMEM (c11885500BT, Gibco) supplemented with 10% fetal bovine serum and 1% penicillin-streptomycin at 37°C in a humidified atmosphere of 5% CO_2_. After overnight culture in serum-free medium, the HK-2 cells were pretreated with 50 uM Xanthohumol followed by 30 mM D glucose for 48 h. Cells were divided into following groups: Control (cells without any treatment); HG (cells were treated with 30 mM D glucose for 48 h); HG+Xanthohumol (cells were pretreated with 50 uM Xanthohumol followed by 30 mM D glucose for 48 h); HG+control-plasmid (cells were pretransfected with control-plasmid followed by 30 mM D glucose for 48 h); HG+SNHG10-plasmid (cells were pretransfected with SNHG10-plasmid followed by 30 mM D glucose for 48 h); HG+Xanthohumol+shRNA-SNHG10 (cells were pretreated with Xanthohumol and shRNA-SNHG10 followed by 30 mM D glucose for 48 h). The cells were collected for subsequent experiments.

### Cell transfection and reagents

The control-plasmid and SNHG10-plasmid were conducted by ELK biotechnology and transfected into HK-2 cells using Lipofectamine^®^ 2000 reagent (11668019, Invitrogen, USA) referring to the manual. After transfection, cells were obtained for following analysis. After 48 h, cells were harvested for the further analysis.

### Biochemical assays

After treatment, Body weights were measured, and blood, serum, urine sample and renal tissues were collected. Urine sample was measured and centrifuged at 2000 *g* for 10 min to acquire supernatant. Urine albumin concentrations were measured using a mouse urine albumin ELISA kit (Cambridge, United States). Blood glucose level was detected using a blood glucose monitor (Boehringer, Germany). The serum creatinine levels were tested using a QuantiChrom Creatinine Assay Kit according to the instructions. The blood urine nitragen (BUN) level was determined using an automatic biochemistry analyser (Beckman Coulter Inc., United States). The renal to body weight ratio was analyzed.

### PAS staining

The 4% paraformaldehyde (80096618, sinoreagent) fixed renal tissues were embedded in paraffin and sectioned at 2∼3 μm of thickness. Then, the sections were dewaxed, hydrated and oxidised by aqueous periodate. Next, the sections were stained with Schiff’s for 30 min and differentiated with 0.5% sodium thiosulfate aqueous solution (20039918, sinoreagent) twice. After washing with tap water, hematoxylin (H9627-25G, Sigma) stained the nuclei for 3 min and 1% hydrochloric acid (10011018, sinoreagent) was used for differentiation. After dehydration and transparency, the slices were sealed using neutral gum for histological analysis.

### Masson staining

The 4% paraformaldehyde (80096618, sinoreagent) fixed renal tissues were embedded in paraffin and sectioned at 2∼3 μm of thickness. Then, the sections were dewaxed, hydrated and stained with Masson Staining Kit (G1006, servicebio) according to the scheme for histological analysis. Collagen fibers were dyed blue, muscle fibers and red blood cells were dyed red, indicating the content of collagen fibers in renal tissue and the degree of fibrosis.

### Immunohistochemistry

Immunohistochemistry is used to detect renal tissue level of α-SMA, FN, Col-I and E-cadherin. The 4% paraformaldehyde (80096618, sinoreagent) fixed renal tissues were embedded in paraffin and sectioned at 5 μm of thickness. The sections were then incubated in citric acid buffer (10007118, sinoreagent) for 10 min and then heated using a microwave oven for antigen repair. After that, the sections were sealed with 5% BSA (4240GR250, Biofroxx) for 30 min, and then incubated overnight at 4°C using α-SMA (BM0002, Boster, 1:400), Fibronectin (Ab268020, Abcam, 1:500), Collagen Ⅰ (14695-1-AP1, Wuhan Sanying, 1:500) or E-Cadherin (20874-1-AP, Wuhan Sanying, 1:200) antibodies. These sections were then cultured in a secondary antibody for 30 min, stained with hematoxylin (H9627-25G, Sigma) and analyzed using a light microscope (CX31, Olympus) according to the scheme.

### ELISA assay

After treatment, renal tissues or HK-2 cells were collected. Then, the levels of inflammatory factors (IL-1β, IL-6 and TNF-α) in renal tissues and the HK-2 cells supernatant were detected using IL-1β ELISA Kit (ELK1270/ELK1271, ELK Biotechnology), IL6 ELISA Kit (ELK1156/ELK1157, ELK Biotechnology), and TNF-α ELISA Kit (ELK1190/ELK1387, ELK Biotechnology) following the instructions, respectively. The optical density (OD) value of each well at 450 nm was detected following the manual.

### qRT-PCR assay

Total RNA was separated from renal tissues or HK-2 cells which used TRIpure Total RNA Extraction Reagent (EP013, ELK, China) according to the synopsis. Total RNA was stored at −80°C. Then cDNA was synthesized using the EntiLink™ 1st Strand cDNA Synthesis Super Mix (EQ031, ELK Biotechnology). Then qRT-PCR was executed using the EnTurbo™ SYBR Green PCR SuperMix (EQ001, ELK Biotechnology). The level of LncRNA SNHG10 was determined using QuantStudio 6 Flex System (Life technologies). Primers sequences are presented in Table 1.

**TABLE 1:** Primer sequences used for PCR.

Gene	Sequences(5′-3′)	
M-ACTIN	Sense	CTG​AGA​GGG​AAA​TCG​TGC​GT
	Antisense	CCA​CAG​GAT​TCC​ATA​CCC​AAG​A
M-SNHG10	sense	GGA​TTC​TTC​CTC​CAG​AAT​GAG​G
	antisense	TAT​GGT​ACC​TGG​AAT​CAG​AGG​ATC
H-ACTIN	sense	GTC​CAC​CGC​AAA​TGC​TTC​TA
	antisense	TGC​TGT​CAC​CTT​CAC​CGT​TC
H-SNHG10	sense	TGG​TTA​TTG​ACT​TCC​TAC​CCA​GC
	antisense	CGG​CTC​CAA​GAC​TAC​AGA​TTA​TG

### CCK-8 assay

CCK-8 assay was conducted using CCK-8 detection kit (C0038, beyotime). After treatment, HK-2 cells were cultured in a 96-well plate. Then CCK-8 reagent (10 μL) was added to each well, followed by incubation at 37°C for 2 h. To evaluate cell viability, a microplate reader (DR-200Bs, Diatek) was used to measure the absorbance at 450 nm.

### Western blot analysis

Total proteins were extracted by HK-2 cells in RIPA buffer (AS1004, ASPEN) supplemented with 1% PMSF (AS1006, ASPEN) and1% phosphatase inhibitors (AS1008, ASPEN). Lysates were centrifuged for 10 min at 4°C, and the supernatants were acquired. Proteins were resolved by SDS-PAGE kit (AS1012, AS1012) and transferred onto PVDF membranes (IPVH00010, Millipore). The membranes were blocked with 5% skimmed milk (AS1033, ASPEN) for 2 h and then cultivated with primary antibodies against α-SMA (ab124964, Abcam, 1:10000), Fibronectin (15613-1-AP, Proteintech, 1:500), Collagen Ⅰ (AF7001, affbiotech, 1:500) or E-Cadherin (#3195, CST, 1:1000) at 4°C overnight. After washing in TBST for three times, the membranes were cultivated with secondary antibodies (AS1107, ASPEN, 1:10000) for 2 h. The protein signals were visualized by ECL detection system reagents (AS1059, ASPEN) and quantified using ImageJ Software.

### Flow cytometry analysis

After treatment, HK-2 cells were plated into 96-well plates for 48 h. After that, the cells were obtained using centrifugal separation technology at 4°C for 5 min and washed with PBS (GNM20012, genomcell). Then cell apoptosis was measured by annexin V-fluorescein isothiocyanate (FITC)/propidium iodide (PI) apoptosis detection kit (AO2001-02P-G, Tianjin Sungene Biotech Co., Ltd) following the instructions. Finally, apoptotic cells were assessed using Flow cytometer (CytoFLEX, Beckman) and analyzed using Kaluza Analysis software.

### Dual-luciferase reporter assay

TargetScan bioinformatics software was applied to predict the binding sites between SNHG10-plasmid and miR-378b. Then the SNHG10-3′UTR, which contains the miR-378b-plasmid binding site or mutated target site were compounded by genomic PCR and cloned into pGL-6-Luc (Promega, United States) to generate the reporter vector SNHG10-wild-type (SNHG10-WT) or SNHG10-mutated-type (SNHG10-MUT). Then HK-2 cells were transfected with SNHG10 wild-type or mutant portion combined with miR-378b-plasmid by applying Lipofectamine 2000 (11668019, Invitrogen) in accordance with the manual. Then the luciferase activity was determined by Dual-Luciferase Reporter Assay System (11668019, beyotime).

### Statistical analysis

The data of three independent experiments were exhibited as the mean ± standard deviation (SD). Statistical analyses was evaluated using GraphPadPrism6.0. Differences were estimated with unpaired Student’s t-test or one-way ANOVA among groups. *P < 0.05, and **P < 0.01 indicated statistically significant difference.

## Results

### Xanthohumol relieved STZ-induced renal injury in DN mice

**FIGURE 1:**
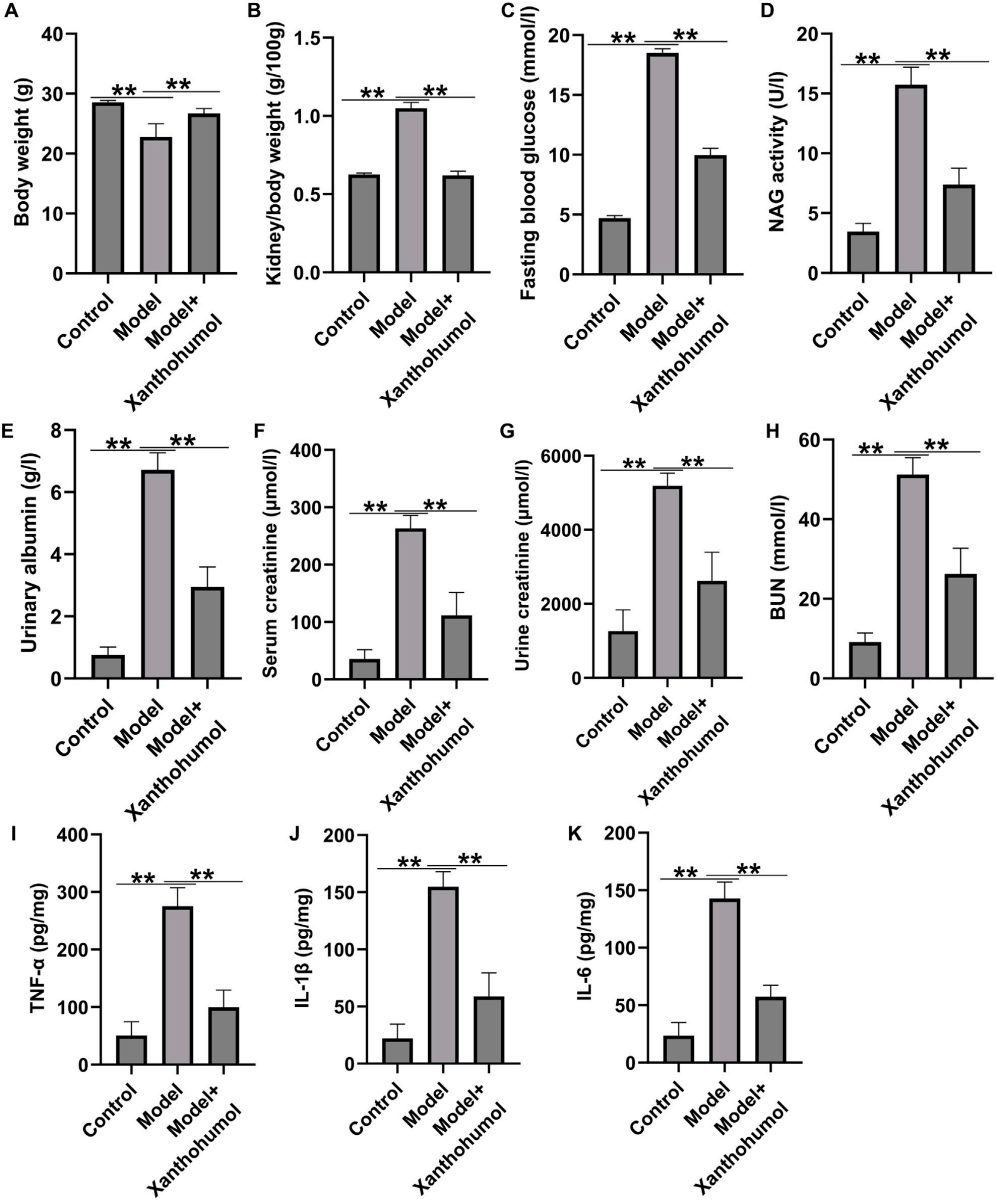
Effects of xanthohumol on renal function in STZ-induced DN mice. STZ-induced DN mice were intraperitoneal injection of 25 mg/kg xanthohumol, followed by the harvest of blood samples and renal tissues. **(A,B)** The body weights and the K/B weight ratio were calculated. **(C)** Blood glucose level was analyzed by Glucose meter. Urinary β-n-acetylglucosaminidase (NAG) activity **(D)**, urinary albumin (ALB) **(E)**, serum creatinine levels **(F)**, urine creatinine levels **(G)**, and blood urine nitragen **(H)** were measured using an automatic biochemistry analyser. **(I–K)** ELISA analysis of serum levels of TNF-α, IL-1β and IL-6. N = 3. **P < 0.01.

To determine the effect of xanthohumol on kidney damage, STZ-induced DN mice were administered with 25 mg/kg xanthohumol. We observed that the body weights were reduced and the ratio of kidney weight/body weight were enhanced obviously in DN mice (Figures 1A,B), and xanthohumol treatment restored these changes. Moreover, STZ treatment led to the remarkable upregulation in the concentrations of blood glucose, NAG activity, urinary albumin, serum creatinine, urine creatinine and BUN (Figures 1C–H), while these influence of STZ were significantly reversed by xanthohumol. ELISA assay manifested that TNF-α, IL-1β, and IL-6 levels (Figures 1I–K) were significantly enhanced in the kidney tissues of STZ-induced DN mice, compared to that in control mice, demonstrating that STZ-induced DN mice had stronger inflammatory responses. However, xanthohumol reduced inflammatory responses in STZ-induced DN mice. These findings demonstrated that xanthohumol alleviated STZ-stimulated kidney injury in DN mice.

### Xanthohumol relieved diabetes-induced renal interstitial fibrosis in the kidneys of DN mice

**FIGURE 2:**
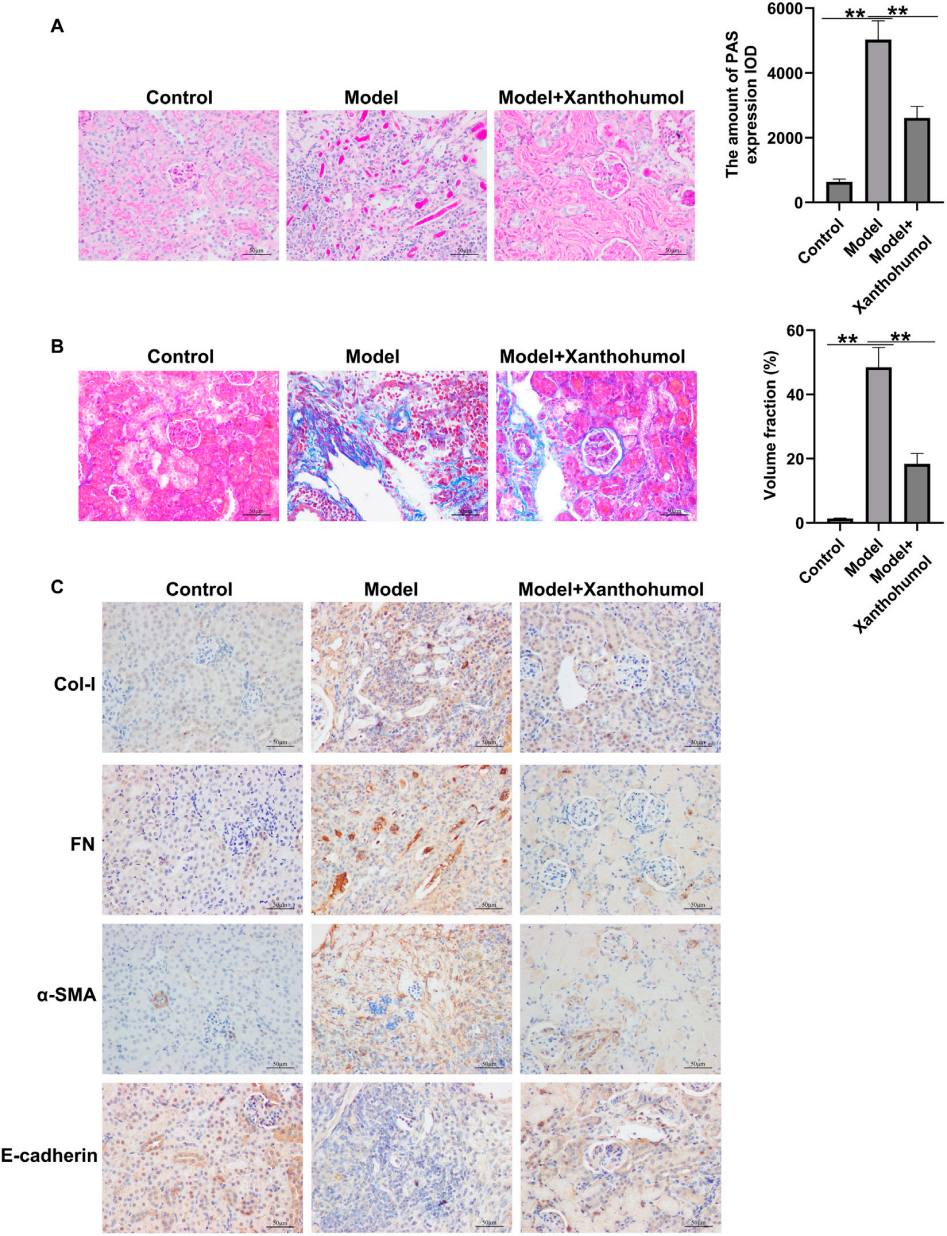
Effects of xanthohumol on pathological changes and renal interstitial fibrosis in the kidneys of DN mice. **(A)** PAS staining of pathological changes of renal tissues. **(B)** Renal tissue sections are stained with Masson and the pathological changes of renal tissues were assessed. Scale: 100 μm. **(C)** IHC analysis of FN, a-SMA, Col-I and E-cadherin in mouse renal tissues. N = 3. **P < 0.01.

To further explore whether xanthohumol relieve renal fibrosis in STZ-induced DN mice, renal fibrosis was assessed by PAS staining and Masson staining and the expression level of fibrotic proteins and epithelial cell markers (FN, a-SMA, Col-I and E-cadherin) were determined by IHC. PAS staining unveiled that glomerular damage and tubulo-interstitial damage were notably increased in STZ-induced DN mice, compared to that in control mice, which was obviously improved by xanthohumol (Figure 2A). Masson staining demonstrated that notably enhanced renal tubulo-interstitial fibrosis in the kidney of DN mice (Figure 2B). Moreover, the expression levels of FN, a-SMA and Col-I were upregulated and E-cadherin was downregulated in DN mice (Figure 2C). However, xanthohumol treatment could remarkably relieve these tubulointerstitial lesions. Together, our findings strongly demonstrated that xanthohumol relieved diabetes-induced renal interstitial fibrosis, thereby blocking DN progression.

### Xanthohumol reversed the expression of lncRNA SNHG10 in the kidney tissues of DN mice

**FIGURE 3:**
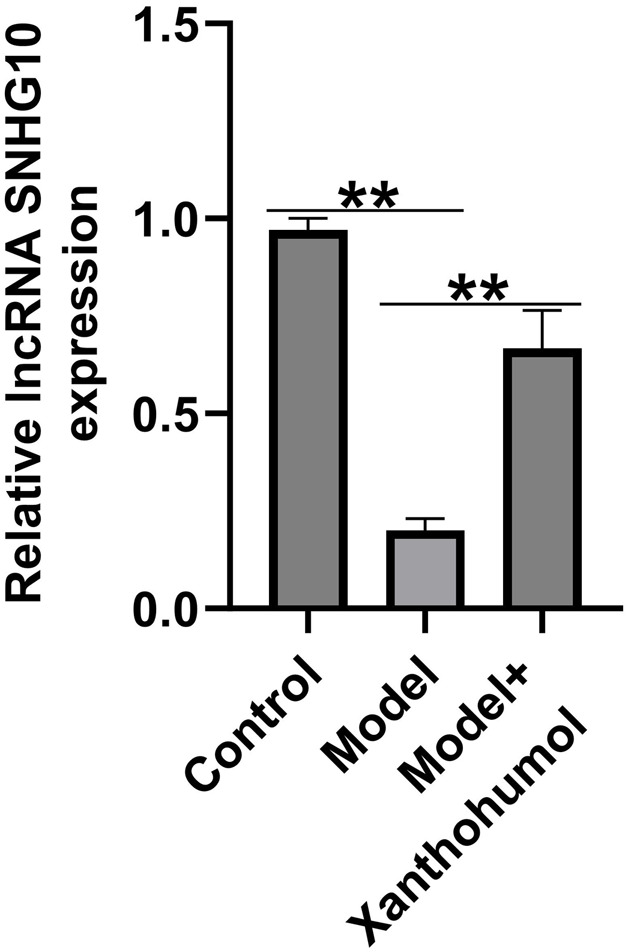
Effects of xanthohumol on the expression of lncRNA SNHG10 in the kidney tissues of DN mice. qRT-PCR analysis was used to measure lncRNA SNHG10 level in renal tissues from DN mice. N = 3. **P < 0.01.

Increasing evidences have highlighted that abnormal expression of lncRNAs are associated with renal fibrosis in renal diseases. To detect the functional roles of lncRNA SNHG10 in the progression of DN, we determined the lncRNA SNHG10 expressions in the renal tissues from DN mice. qRT-PCR analysis revealed that lncRNA SNHG10 was downregulated in the renal tissues of DN mice as compared to that in control groups, and this inhibition was reversed by xanthohumol treatment (Figure 3). Our data indicated that abnormal expression of lncRNA SNHG10 may be linked with the pathogenesis of DN.

### Xanthohumol regulated HK-2 cells viability, apoptosis, renal interstitial fibrosis and inflammatory response by regulating lncRNA SNHG10

**FIGURE 4:**
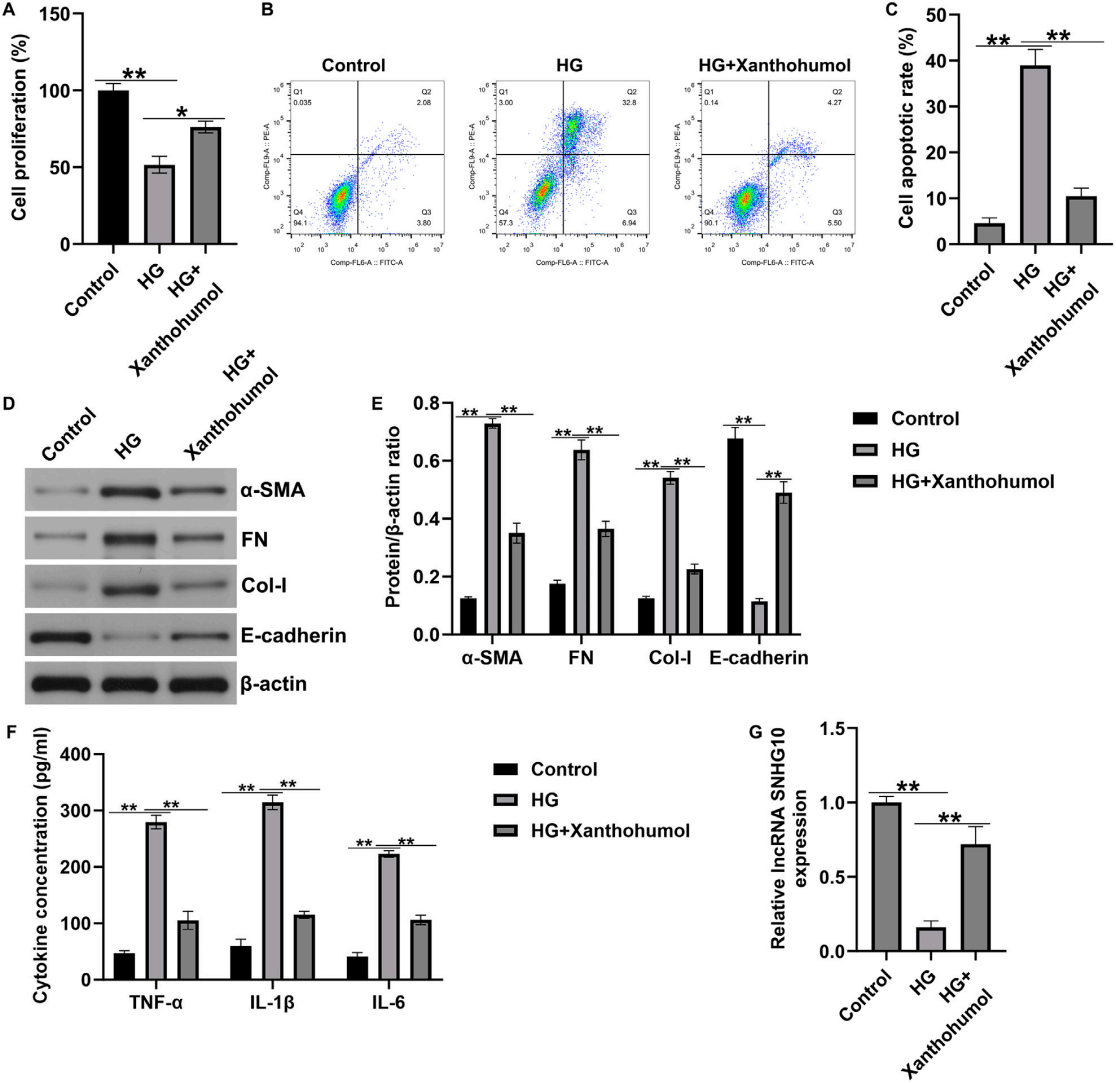
Effects of xanthohumol on cells viability, apoptosis, renal fibrosis and inflammatory response in high glucose-induced HK-2 cells. HK-2 cells were exposed to high glucose and treated with 50 uM xanthohumol. **(A)** CCK-8 assay of cell viability. **(B)** Cell apoptosis was detected by Flow cytometry assay. **(C)** Quantification of apoptotic cells. **(D,E)** The expression of fibrotic proteins and epithelial cell markers, including FN, a-SMA, Col-I and E-cadherin, were determined by Western blot. **(E)** Quantification of FN, a-SMA, Col-I and E-cadherin expression relative to β-action. **(F)** The secretion of TNF-α, IL-1β and IL-6 were determined using ELISA. **(G)** The levels of lncRNA SNHG10 in HK-2 cells were measured by qRT-PCR analysis. N = 3. *P < 0.05, and **P < 0.01.

Moreover, an *in vitro* DN model was established by simulating renal tubular epithelial cells injury with high glucose, then the HK-2 cells were treated with 50 uM xanthohumol. CCK-8 assay and flow cytometer analysis were applied to determine HK-2 cells viability and apoptosis. As presented in Figures 4A–D-glucose suppressed the HK-2 cells viability and stimulated more apoptotic cells. However, these findings were partly reversed by xanthohumol. We also determined the expression of fibrotic proteins and epithelial cell markers, including FN, a-SMA, Col-I and E-cadherin, were determined by Western blot. Our data suggested that the expression of α-SMA, FN and Col-I were higher in high glucose-induced HK-2 cells than that in Control group. Furthermore, E-cadherin was downregulated in high glucose-induced HK-2 cells, compared with untreated cells. Nevertheless, we observed the opposite results in 50 uM xanthohumol treated cells (Figures 4D,E). To explore the effect of xanthohumol on inflammatory response, we detected TNF-α, IL-1β, and IL-6 levels in culture supernatant of HK-2 cells by ELISA. Our data demonstrated that high glucose notably promoted the secretion of inflammatory factor, and this promotion was inhibited by xanthohumol treatment (Figure 4F). In addition, results from Figure 4G suggested that xanthohumol enhanced the mRNA level of lncRNA SNHG10 in high glucose-induced HK-2 cells, as opposed to Control group. Our data confirmed that xanthohumol ameliorated diabetic nephropathy through suppression of renal fibrosis and inflammatory response by regulating lncRNA SNHG10 levels in the pathogenesis of DN.

### SNHG10-plasmid relieved STZ-induced renal injury in DN mice

**FIGURE 5:**
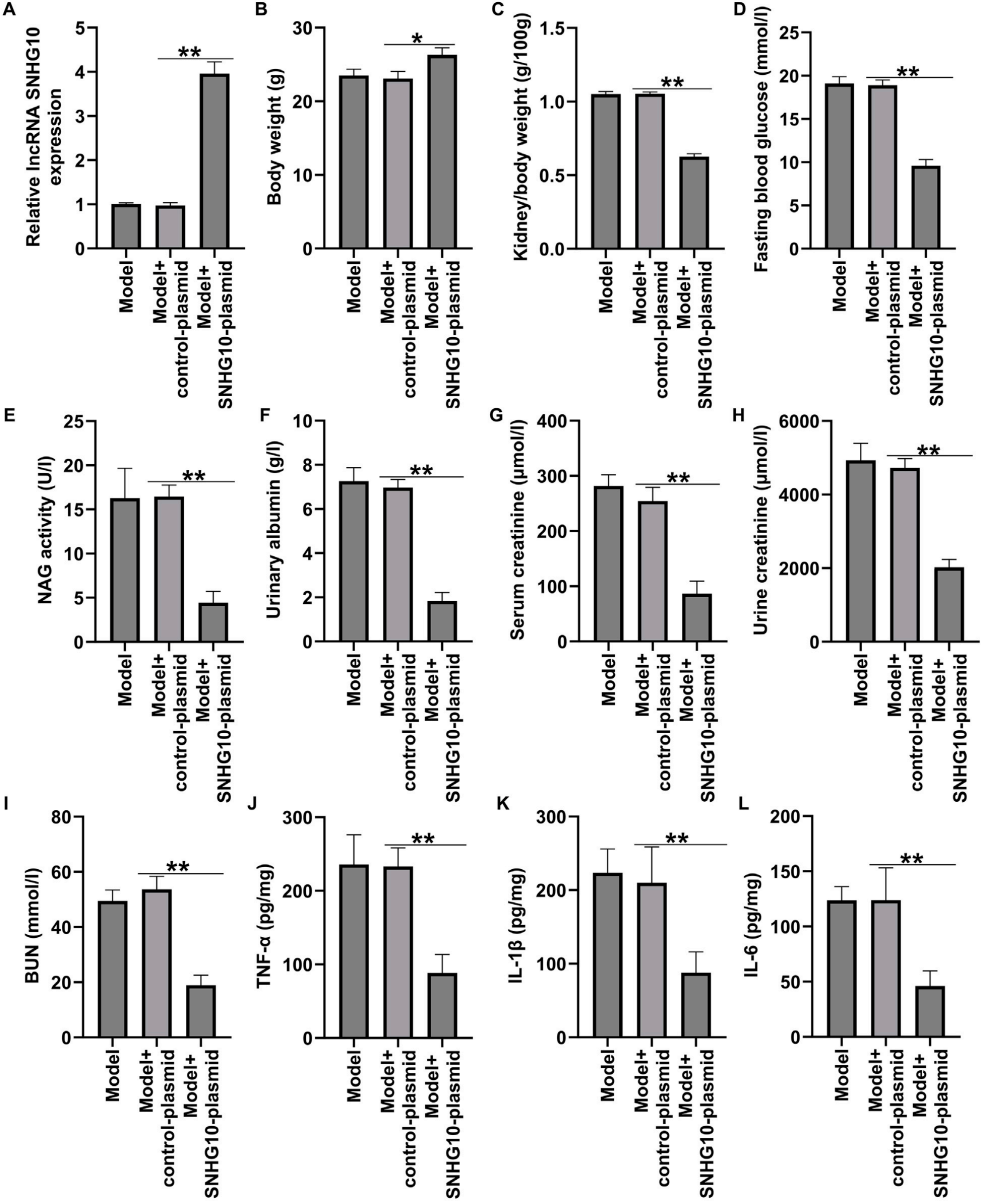
Effects of SNHG10-plasmid on renal function and inflammatory response in STZ-induced DN mice. STZ-induced DN mice were intraperitoneal injection of SNHG10-plasmid, followed by the harvest of blood samples and renal tissues. **(A)** The levels of lncRNA SNHG10 were measured by qRT-PCR analysis. **(B)** Body weights, **(C)** kidney weight/body weight ratio, **(D)** blood glucose concentrations, **(E)** NAG activity, **(F)** 24-h urinary albumin, **(G)** serum creatinine levels, **(H)** urine creatinine levels, and **(I)** blood urine nitragen in STZ-induced DN mice were determined by automatic biochemistry analyser. ELISA analysis of serum levels of TNF-α **(J)**, IL-1β **(K)** and IL-6 **(L)**. N = 3. *P < 0.05, and **P < 0.01.

Our results have revealed the relationship between DN occurrence and lncRNA SNHG10. To further explain the function of lncRNA SNHG10 in the pathogenesis of DN. STZ-induced DN mice were intraperitoneally injected with control-plasmid or SNHG10-plasmid and we observed that lncRNA SNHG10 was upregulated in the renal tissues from DN mice, compared with Control and control plasmid group (Figure 5A). Our results further confirmed that SNHG10-plasmid relieved STZ-induced renal injury in DN mice, as verified by increased body weights, decreased kidney weight/body weight ratio, blood glucose concentrations, NAG activity, 24-h urinary albumin, serum creatinine, urine creatinine and BUN (Figures 5B–I), compared with control plasmid group. After that, to delineate whether SNHG10-plasmid may reduce the inflammatory response in DN renal tissue, we detected the secretion of inflammatory factors, including TNF-α, IL-1β, and IL-6 levels. As documented in Figures 5J–L, the secretion levels of inflammatory factors in the SNHG10-plasmid group were visibly lower than those in the control plasmid group. Our findings demonstrated that upregulation of SNHG10 alleviated STZ-stimulated kidney injury in DN mice.

### Xanthohumol ameliorated DN through suppression of renal interstitial fibrosis by regulating SNHG10

**FIGURE 6:**
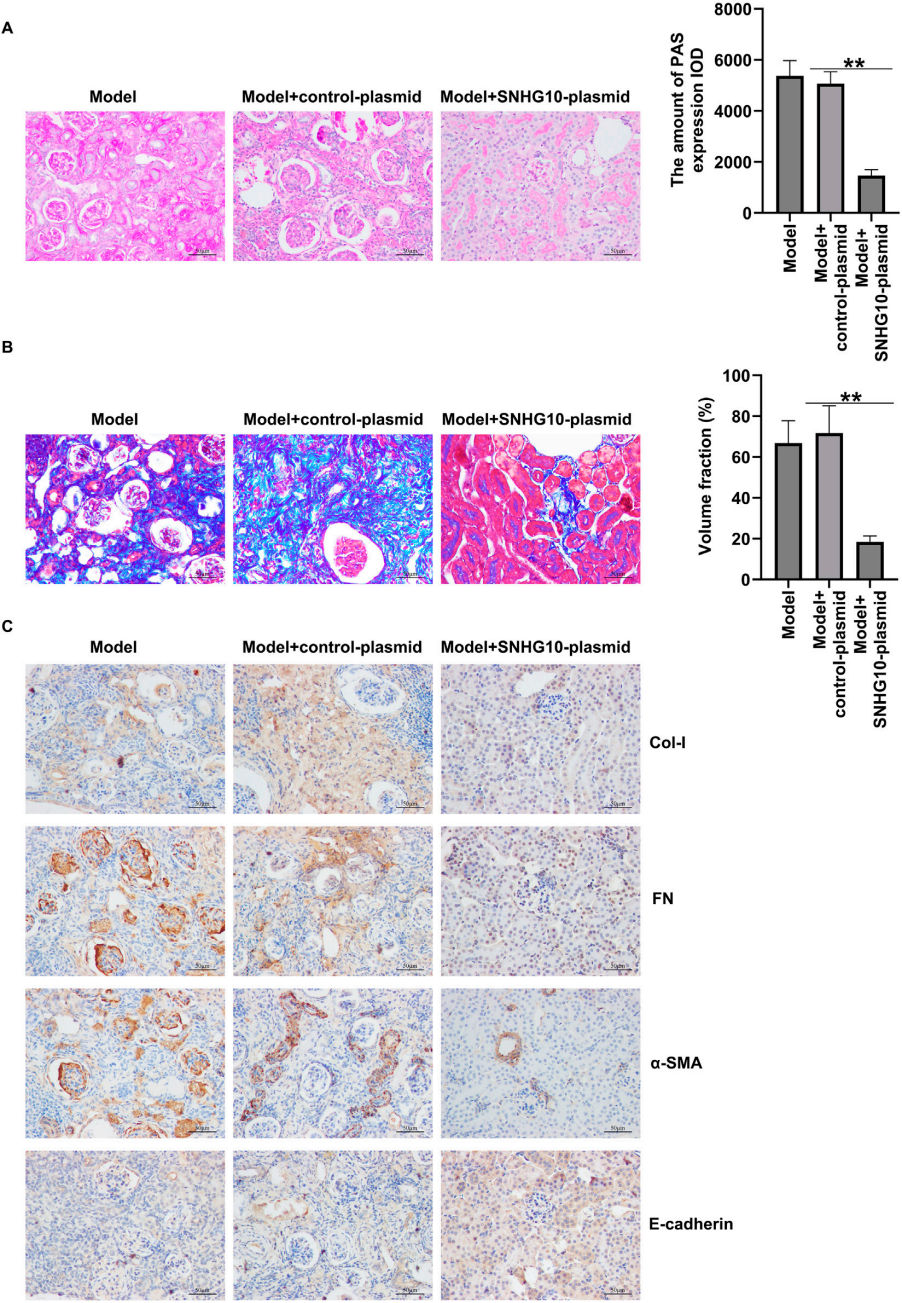
Effects of SNHG10-plasmid on renal interstitial fibrosis in the kidneys of in STZ-induced DN mice. **(A)** Pathological changes of renal tissues were detected by PAS staining. **(B)** Pathological changes of renal tissues were detected by PAS staining. Scale: 100 μm. **(C)** IHC analysis of FN, a-SMA, Col-I and E-cadherin in mouse renal tissues. scale: 100 μm. N = 3. *P < 0.05, and **P < 0.01.

Next, PAS and Masson staining unveiled that upregulation of SNHG10 remarkably relieved glomerular damage and renal tubulo-interstitial fibrosis in the kidney of DN mice (Figures 6A,B). Further IHC staining indicated the decreased FN, a-SMA and Col-I expression, as well as enhanced E-cadherin level in the renal tissues of SNHG10-plasmid treated DN mice, as opposed to control plasmid group (Figure 6C). Our data demonstrated that xanthohumol suppressed renal interstitial fibrosis of DN mice by regulating SNHG10.

### Xanthohumol ameliorated DN through suppression of renal interstitial fibrosis and inflammatory response by regulating SNHG10

**FIGURE 7:**
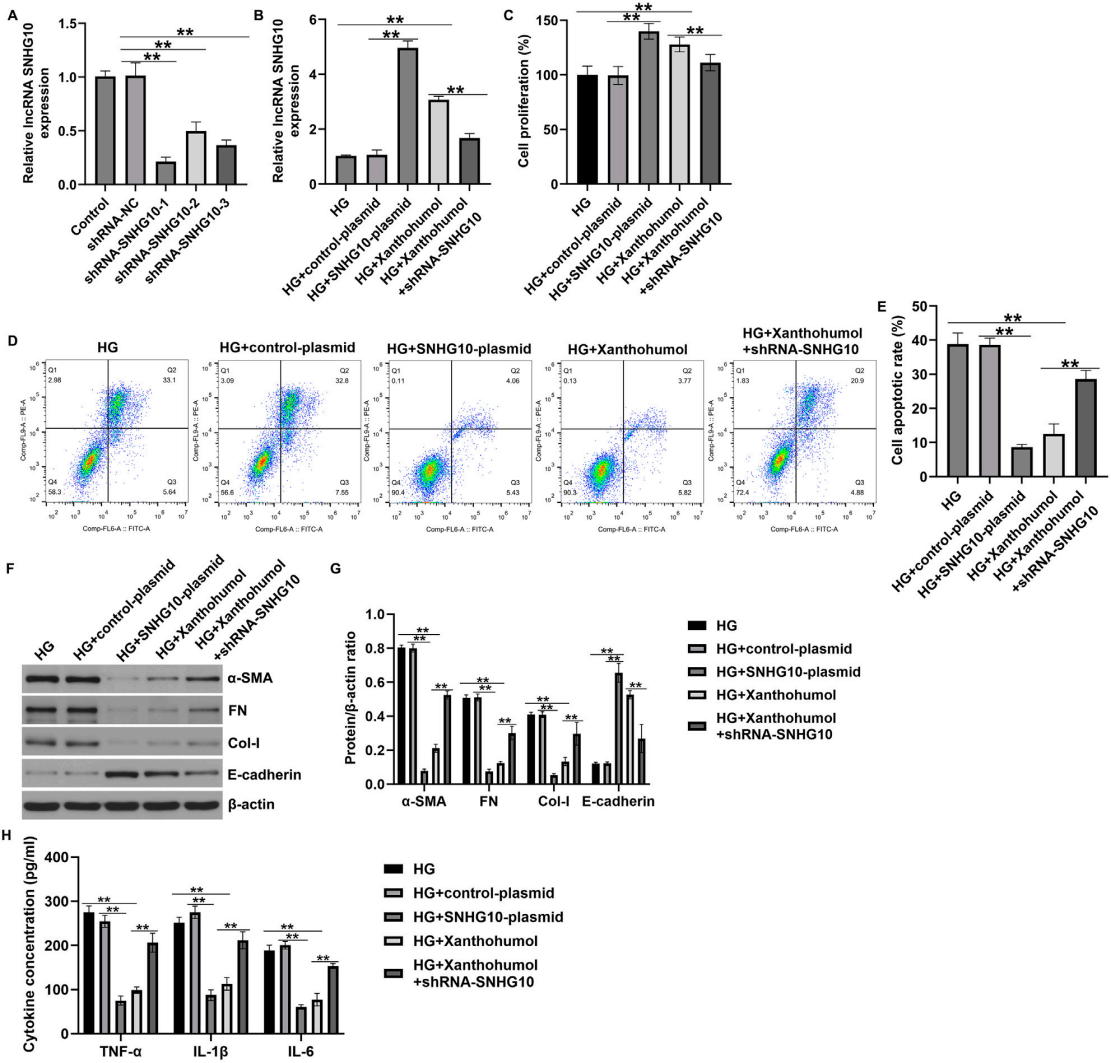
Downregulation of SNHG10 reversed the effects of xanthohumol on renal fibrosis and inflammatory response HK-2 cells. The high glucose-induced HK-2 cells were treated with xanthohumol or SNHG10-plasmid, followed by shRNA-SNHG10 stimulation. **(A,B)** The levels of SNHG10 in different groups were determined by qRT-PCR analysis. **(C)** CCK-8 assay of cell viability. **(D)** Cell apoptosis was detected by Flow cytometry assay. **(E)** Quantification of apoptotic cells. **(F,G)** Detection of fibrotic proteins and epithelial cell markers using Western blot assay. **(H)** ELISA analysis of serum levels of TNF-α, IL-1β and IL-6. N = 3. **P < 0.01.

To further explain the regulation of lncRNA SNHG10 and xanthohumol in the development of DN, we designed three shRNA to silence the expression of SNHG10 in HK-2 cells. RT-qPCR analysis confirmed the knockout efficiency of HK-2 cells, and we observed that shRNA-SNHG10-1 knockout decreased most significantly SNHG10 mRNA levels in HK-2 cells, compared with the other two plasmids (Figure 7A). Thus, shRNA-SNHG10-1 was selected for subsequent cell function experiments. Results from Figure 7B revealed that SNHG10 was upregulated in high glucose-induced HK-2 cells after SNHG10-plasmid or xanthohumol treatment, while this promotion was inhibited by 50 μM xanthohumol+shRNA-SNHG10. Further CCK-8 and FCM assay revealed that SNHG10-plasmid or xanthohumol promoted high glucose-induced HK-2 cells viability and reduced cells apoptosis (Figures 7C–E). However, we observed the opposite data in 50 μM xanthohumol+shRNA-SNHG10 treated cells. In addition, Downregulation of SNHG10 reversed the effects of xanthohumol or SNHG10-plasmid on renal interstitial fibrosis and inflammatory response in high glucose-induced HK-2 cells, as confirmed by enhanced α-SMA, FN, Col-I, TNF-α, IL-1β, and IL-6 levels, as well as suppressed E-cadherin expression (Figures 7F–H). Our findings demonstrated that xanthohumol ameliorates DN through suppressing renal interstitial fibrosis and inflammatory response by regulating SNHG10.

### MiR-142-3p was a target of lncRNA SNH 10

**FIGURE 8:**
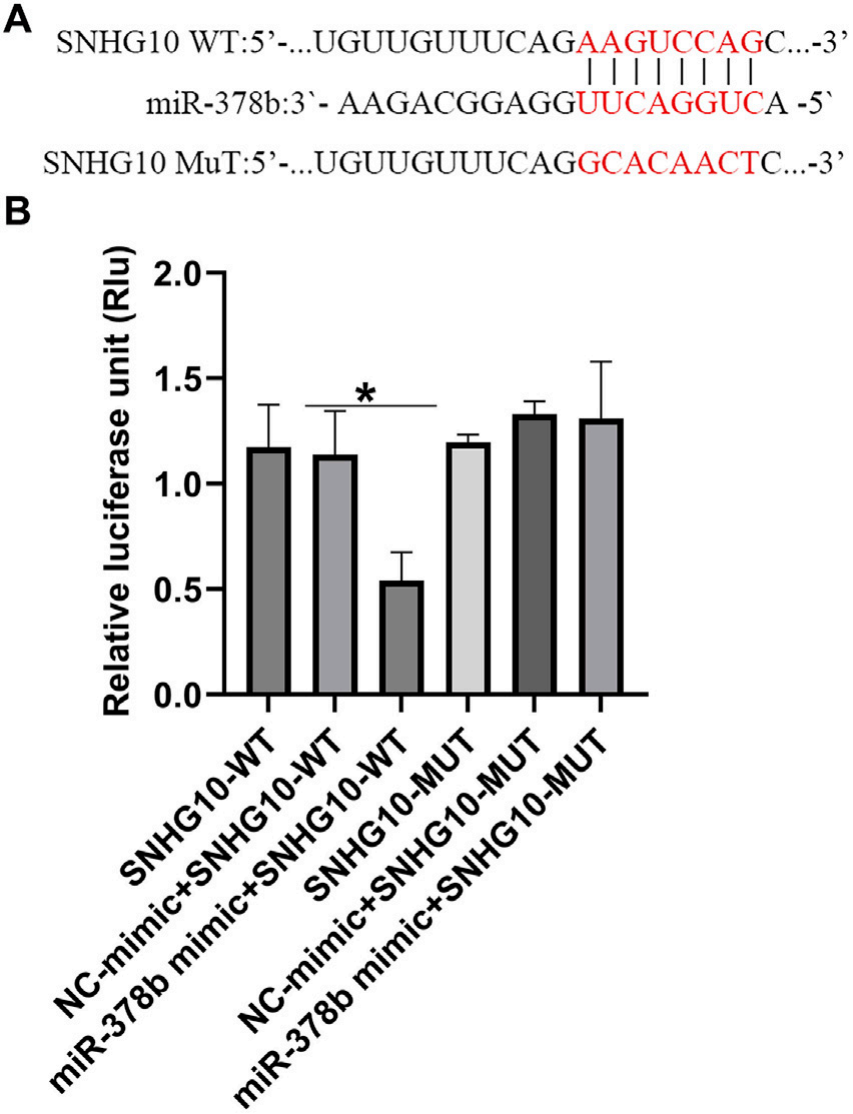
MiR-378b directly targets lncRNA SNHG10 **(A)** The conserved target sites of miR-378b binding to SNHG10 3′-UTR were shown. **(B)** Luciferase activities in 293T cells transfected with WT- SNHG10 or MUT-SNHG10 and miR-378b mimic. N = 3. *P < 0.05.

To investigate the possible molecular mechanism of lncRNA SNHG10 in regulating DN procession, TargetScan (http://www.targetscan. org) was applied to identify the latent targets of lncRNA SNHG10. As presented in Figure 8A, miR-378b contained complementary binding sites to the 3′-UTR of lncRNA SNHG10. To reveal the direct binding between lncRNA SNHG10 and miR-378b, SNHG10 (WT) and SNHG10 (MUT) luciferase reporters containing miR-378b NC or miR-378b mimics binding sites were individually constructed. The luciferase reporter assay revealed that miR-378b mimics prominently reduced the luciferase activity of lncRNA SNHG10 (WT) reporter in 293 T cells, while overexpression of miR-378b displayed little effect on luciferase activity in MUT-lncRNA SNHG10 transfected cells (Figure 8B). Our findings suggested that miR-378b directly targets lncRNA SNHG10.

## Discussion

DN, a major complication of diabetes mellitus, causes severe renal tubular and interstitial damage, and eventually leads to death (Cui et al., 2023). In recent years, it has been found that a variety of factors such as reactive oxygen species and high glucose can lead to diabetic kidney injury, which in turn leads to renal tubulointerstitial inflammation and fibrosis and promotes the progression of DN (Zhuang et al., 2022). With the in-depth study of DN, the role of tubulointerstitial lesions and interstitial fibrosis in the progression of DN has been widely concerned (Xu et al., 2024). However, the treatment of DN still lacks effective drugs, and more effective therapies need to be developed to improve the health of DN patients. Besides, screening natural small molecule drugs that have been applied in the market or clinical trials has high safety for the treatment of DN (Lv et al., 2023; Ma et al., 2021a). Xanthohumol, which has a variety of pharmacological activities (Zhu et al., 2023; Liu et al., 2020; Niederau et al., 2022), has been shown to have a certain protective effect in DN (Li et al., 2023). In this study, we studied the specific role and molecular mechanism of xanthohumol in DN both *in vivo* and *in vitro*.

STZ-treated DN mice models and HG-induced HK-2 cell models have been widely used to elucidate the pathogenesis of DN (Wang et al., 2022; Wang and Lu, 2022; Liu et al., 2023; Wang et al., 2024). In our study, DN mice model and HG-induced HK-2 cell model were used to investigate the effects of xanthohumol on DN. Our data confirmed that xanthohumol effectively improves DN-stimulated kidney structural and functional abnormalities through reducing albuminuria, glomerular and tubular injury, renal inflammation and interstitial fibrosis. We next investigated whether xanthohumol plays a protective role in HG-induced renal tubular epithelial cells. We observed that HG treatment significantly reduced cell viability and increased apoptosis rate, which is consistent with the results of other investigations (Wang et al., 2022; Wang and Lu, 2022). Furthermore, it has been shown that the exposure of HK-2 cells to high glucose can result in inflammation (Wang et al., 2022). And the stimulation of HG induces EMT and fibrosis in renal tubules, leading to persistent albuminuria, which is a characteristic of DN (Liu et al., 2023; Wang et al., 2024). These were verified in this report, as the fibrosis index and inflammatory factor levels were increased in the high glucose-induced HK-2 cells. Nevertheless, we observed the opposite finings in xanthohumol treated group. Our observations demonstrated that xanthohumol protects renal tubular epithelial cells from HG-induced injury through its anti-apoptotic and anti-inflammatory effects.

Accumulating evidences suggested that lncRNAs play a key role in various biological processes by recruiting chromatin modification complexes to their nuclear promoters to regulate the expression of target genes (Xu et al., 2023). Several studies have reported that lncRNAs are associated with the pathogenesis of DN. For example, Zhang et al. have suggested the biological effects of lncRNA CASC11 on aggravating DN by regulating FoxO1 (Zhang et al., 2023a; b). Moreover, report from Zhao et al. demonstrated that lncRNA MSC-AS1 aggravates DN by regulating the miR-325/CCNG1 axis (Zhao et al., 2022). LncRNA SNHG10 has been reported to be a participant in multiple tumor progression (Aini et al., 2022; Zhu et al., 2020). However, its potential function and mechanism in DN remain unclear. In this study, we confirmed the lower expression of SNHG10 in the renal tissues of DN mice and HG-stimulated HK-2 cells, and this inhibition was reversed by xanthohumol treatment. Thus, we hypothesized that SNHG10 is involved in the development and progression of DN. To further explain the function of lncRNA SNHG10 in the pathogenesis of DN, we investigated the roles of SNHG10-plasmid in DN mice model and in HG-induced HK-2 cells. Our data suggested that upregulation of SNHG10 alleviated STZ-stimulated kidney injury in DN mice and HG induced HK-2 cell injury. Meanwhile, we found that SNHG10 inhibition reversed the effects of xanthohumol on HK-2 cells, indicating that xanthohumol ameliorates DN through suppressing renal interstitial fibrosis and inflammatory response by regulating SNHG10.

LncRNAs can act as miRNA sponges, reducing their regulatory effect on mRNAs (Paraskevopoulou and Hatzigeorgiou, 2016). LncRNAs-mediated regulation of miRNAs affecting DN and propose novel molecular-level therapeutic strategies for DN (Tian et al., 2025; Li G. et al., 2021). In recent years, wide attentions have been paid to the functions of lncRNA-miRNA pairs in renal pathophysiology, especially in DN. For example, Yang et al. revealed that lncRNA MALAT1 promotes DN progression via miR-15b-5p/TLR4 signaling axis (Yang et al., 2022). Moreover, Gu et al. demonstrated that TUG1 overexpression alleviates kidney injury in DN mice and decreases the inflammatory response and fibrosis of high glucose-stimulated HK-2 cells via miR-145-5p/DUSP6 axis (Gu et al., 2019). To further investigate the underlying mechanism of SNHG10 in the DN progression, we searched for potential targets of SNHG10. In our report, bioinformatics and luciferase reporter analysis indicated that miR-378b was a potential target of SNHG10. MiR-378b was evidenced to be a vital regulator in the progression of diseases, including lung cancer (Wang et al., 2020), hepatic steatosis (Li N. et al., 2020) and hypoxic-ischemic brain damage (Li H. et al., 2021). Besides, Xiong et al. suggested the protective function of miR-378 in the ischemia-reperfusion injury during renal transplantation and interstitial fibrosis (Xiong et al., 2020). Given the regulatory role of miR-378b in insulin resistance (Li et al., 2020a) and organ fibrosis (Hyun et al., 2016), we hypothesize that SNHG10 may play a role in DN by regulating miR-378b. However, the specific role and molecular regulatory mechanism of miR-378b in DN still need to be further explored.

In conclusion, the present study demonstrated that xanthohumol inhibited renal tubulointerstitial fibrosis by regulating the SNHG10/miR-378b pathway, thereby alleviating DN. Our study further clarified the mechanism of xanthohumol in DN and provided new therapeutic drugs for DN. In the future, we will further explore the effect of miR-378b on DN, hoping to further clarify the protective mechanism of xanthohumol on DN in clinical application.

## Data Availability

The raw data supporting the conclusions of this article will be made available by the authors, without undue reservation.
